# Mast cell sarcoma: new cases and literature review

**DOI:** 10.18632/oncotarget.11812

**Published:** 2016-09-01

**Authors:** Jilliana Monnier, Sophie Georgin-Lavialle, Danielle Canioni, Ludovic Lhermitte, Michael Soussan, Michel Arock, Julie Bruneau, Patrice Dubreuil, Christine Bodemer, Marie-Olivia Chandesris, Olivier Lortholary, Olivier Hermine, Gandhi Damaj

**Affiliations:** ^1^ Service de Médecine Interne, Hôpital Tenon, Université Paris VI, Assistance Publique-Hôpitaux de Paris (AP-HP), Paris, France; ^2^ Centre de Référence des Mastocytoses, Faculté de Médecine et AP-HP Necker-Enfants Malades, Paris, France; ^3^ Laboratoire d’Anatomie-Pathologie, Université Paris Descartes, Paris Cité, Faculté de Médecine et AP-HP Necker-Enfants Malades, Paris, France; ^4^ Département de Médecine Nucléaire, Hôpital Avicenne, AP-HP et Université Paris 13, Bobigny, France; ^5^ Laboratoire d’Hématologie, Groupe Hospitalier Pitié-Salpêtrière, AP-HP, Paris, France; ^6^ LBPA CNRS UMR8113, Ecole Normale Supérieure de Cachan, Cachan, France; ^7^ Inserm, U1068, CRCM, [Signaling, Hematopoiesis and Mechanism of Oncogenesis], Institut Paoli-Calmettes, Marseille, Aix-Marseille Univ, CNRS UMR7258, Marseille, France; ^8^ Service de Dermatologie de l’Hôpital Necker Enfants-Malades, AP-HP, Paris, France; ^9^ Service d’hématologie Adulte, Université Paris Descartes et Institut Imagine, Hôpital Necker-Enfants Malades, Paris, France; ^10^ Institut d’Hématologie de Basse Normandie, Centre Hospitalier Universitaire, Caen, France; ^11^ Microenvironnement Cellulaire et Pathologies, Normandie Univ, Unicaen, MILPAT, Caen, France

**Keywords:** mast cell, mastocytosis, mast cell sarcoma, KIT mutations, targeted therapies

## Abstract

Mast cell sarcoma (MCS) is a rare form of mastocytosis characterized by the presence of solid tumor(s) comprising malignant mast cells that harbor destructive infiltration capability and metastatic potential. Here, we present an extensive literature review and report on 23 cases of MCS, including 3 new cases from the French National Reference Center for Mastocytosis. From our analysis, it appears that MCS can occur at any age. It can manifest *de novo* or, to a lesser extent, may evolve from a previously established mastocytosis. Bone tumor is a frequent manifestation, and symptoms of mast cell activation are rare. Histological diagnosis can be difficult because MCS is frequently composed of highly atypical neoplastic mast cells and can thus mimic other tumors. Unexpectedly, the canonical *KIT* D816V mutation is found in only 21% of MCS; therefore, complete *KIT* gene sequencing is required. The prognosis of patients with MCS is poor, with a median survival time of less than 18 months, and progression to mast cell leukemia is not unusual. Because conventional chemotherapies usually fail, the role of targeted therapies and bone marrow transplantation warrants further investigation in such aggressive neoplasms.

## INTRODUCTION

Mast cell neoplasms comprise a clinically and biologically heterogeneous group of disorders characterized by abnormal accumulation of atypical mast cells (MCs) in one or several organs [1].

Mast cell sarcoma (MCS) is an exceedingly rare and malignant form of solid tumor composed of highly atypical MCs and characterized by destructive infiltration and metastatic potential. Clinical presentation is variable and proper diagnosis may be very difficult due to multiple differential diagnoses [2]. Genetic features are barely known and therapeutic options are limited. The prognosis of patients is dismal, and the tumor is most often fatal within a few months [2, 3]. The majority of the available information on MCS is based only on case reports. For this reason, we aimed to review all published data on MCS and, in addition, included 3 previously unpublished French cases. Therefore, this review includes 23 cases of SM: 20 cases from the literature and three new French cases from the French National Reference Center for Mastocytosis. The goal of this extended review is to help better define the clinical and biological presentation of MCS as well as their prognostic profiles and outcomes.

## EPIDEMIOLOGY

MCS was described as early as 1986 by Horny et al [4]. It is an exceedingly rare subcategory of mastocytosis and accounts for less than 0.5% of all French cases of mastocytosis reported in the CEREMAST database.

The median patient age at diagnosis was 41 years (range: 1-77) with a slight predominance of females (13 females/10 males). After excluding 5 pediatric cases, the median age of the adult group at diagnosis was 55 years (range: 19-77). The disease appears to be sporadic, and only one familial case has been described [3]. *De novo* MCS was the most frequent presentation, representing 91% of the cases (21 patients). Only two patients (9%) had a history of cutaneous mastocytosis, and they were considered to have secondary MCS. The age of onset of cutaneous lesions in these two patients were 1 and 10 years, respectively. These two latter cases are of clinical interest and highlight the need for providing medical advice if new symptoms appear and for long-term follow-up of patients with mastocytosis.

## CLINICAL MANIFESTATIONS

**Figure 1:**
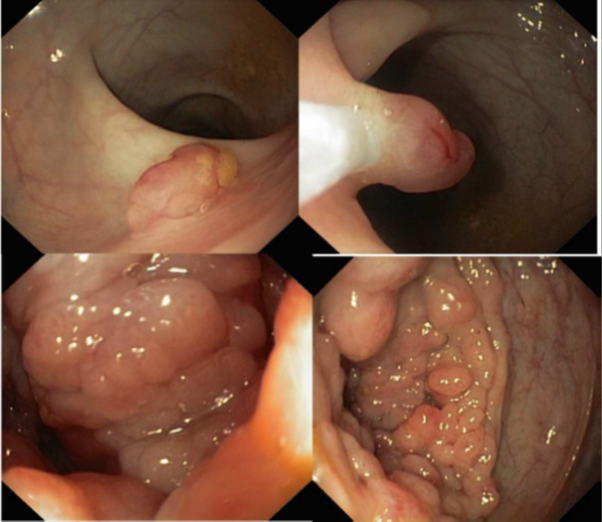
Endoscopic findings of a gastro-intestinal mast cell sarcoma (# 23) There is a burgeoning polylobar tissue lesion of the right colon (arrow).

MCAS were observed in nearly one-third (7/23, 30%) of patients. These symptoms included flushing, fever, malaise, diarrhea and tachycardia. The most common organ involved was bone (78%), with masses found in the thoracic vertebrae, pelvis, tibia, femur, ankle and skull; followed by the gastro-intestinal tract (35%) (Figure 1); lymph nodes (30%); skin (30%); spleen (26%); and liver (22%) (Table 1). Sarcoma may also be localized in the uterus (*n* = 1), testicles (*n* = 1), oropharyngeal tract (including the lips, ears and larynx (n = 3)) and eyes (*n* = 1). Of note, all 5 pediatric cases had bone localization (Table 1), and 80% (*n* = 4) of these had cephalic bone localization, including the temporal bone, external auditory tract and ear. This could suggest that careful clinical long-term follow-up of children and young adults with mastocytosis is necessary and may include examinations of the head, ears and skull for the children.

**Table 1:** Clinical and biological characteristics of MCS patients as obtained from the literature review

	All MCS	MCS with pediatric mastocytosis	Adult MCS
	*n* = 23	*n* = 5	*n* = 18
**Age at diagnosis of MCS (years) median (range)**	41 (1 – 77)	8 (1 – 15)	55 (19 – 77)
**Gender (Female/Male)**	13/10	3/2	10/8
**Familial mastocytosis, *n* (%)**	1/23 (4.3)	1/5 (0.2)	0/18 (0)
**Median time from symptoms to diagnosis (range, months)**	15 (0-21)	0,2 (0 – 1)	20 (0 –216)
**History of mastocytosis, *n* (%)**	2/23 (9)	0/5 (0)	2/18 (11)
**Organ Involvement, *n* (%)**			
Bones	18 /23 (78)	5/5 (100)	13/18 (72)
Digestive tract	8/23 (35)	0/5 (0)	8/18 (45)
Lymph nodes	7/23 (30)	0/5 (0)	7/18 (38)
Skin (UP)	7/23 (30)	0/5 (0)	7/18 (38)
Splenomegaly	6/23 (26)	1/5 (20)	5/18 (28)
Hepatomegaly	5/23 (22)	1/5 (20)	4/18 (22)
**MCAS, *n* (%)**	7/23 (30)	1/5 (20)	6/18 (33)
*KIT* mutation^*^, *n*	14	3	11
WT *KIT, n (%)*	7 (50)	2 (67)	5 (46)
other mutation, *n (%)*	4 (29)	1 (33)	3 (27)
D816V *KIT* mutation*, n (%)*	3 (21)	0	3 (27)
**Laboratory data, median (range)**			
Tryptase (ng/L)	236 (8,6 – 900)	145 (34 – 200)	233 (8,6 – 900)
Hemoglobin (g/dL)	10,6 (8 – 14)	11,6 (9,6 – 14)	11,25 (8 – 14)
Platelets (G/L)	309 (30 – 486)	300 (300 – 300)	300 (30 − 486)
Leukocytes (G/L)	9,3 (2,8 – 15)	12 (12 – 12)	7,8 (2,8 − 15)
**Outcome**			
MCL evolution, n (%)	7/23 (30)	2/5 (40)	5/18 (28)
Death, n (%)	12/20 (60)	2/2 (100)	10/18 (55)
Median survival time; (range; months)	17 (1-45)	22,2 (12-45)	15,5 (1– 40)

MCAS; symptoms of MC activation (including flushing, fever, malaise, diarrhea and tachycardia); MCL: mast cell leukemia; MCS: mast cell sarcoma; UP; urticaria pigmentosa; WT: wild-type;

* Asp 822 Lys (exon 17), Leu 799 Phe (exon 17), Val 560 Gly (exon 11), Del D419 (exon 8)

## LABORATORY CHARACTERISTICS

### Biological features

The median hemoglobin level, platelet and leukocyte counts were 10.6 g/dl (range: 8-14),

309 G/L (range 30-486) and 9.3 G/L (range 2.8-15), respectively. Serum tryptase levels were increased in the majority of cases, with a median level of 236 ng/L (range: 8.6-900) (Table 1);

### Histological findings and immunohistochemistry (IHC) of MCS

Mast cells in MCS rarely share histologic features encountered in systemic mastocytosis, such as spindle-shaped MCs and MCs with an eccentric oval nucleus or hypogranulated cytoplasm. Indeed, in MCS, MCs presented more frequently as medium to very large pleomorphic or sometimes epithelioid cells, as having oval or bilobed nuclei, or even as multinucleated cells. Granules can be observed in abnormal MCs using Giemsa stain. In some cases, MCs were highly heterogeneous and differed from site to site within the same patient. Tumor infiltration by eosinophils is frequently encountered and may be marked.

Highly atypical MCs found in MCS usually expressed tryptase, KIT (CD117) and CD68. However, CD25 was haphazardly expressed. Indeed, sarcomatous MCs were negative for CD25 in 25% of the cases and negative for CD2 in 48% of the cases. CD30, which has been suggested to be a marker for SM aggressiveness, appeared to be rarely expressed in MCS.^5^ Only 3 of the 11 patients tested (27%) were positive for CD30. A positive CD30 test may mislead physicians to an inaccurate diagnosis of ALCL. However, this marker is a potential target of brentuximab (a therapy that remains to be evaluated) and should be systematically tested for in patients.Finally, Ki67 expression was variable (6 of 14 patients (43%) tested were positive for Ki67 expression).

These highly atypical MCs are often misdiagnosed with multiple differential diagnoses, including carcinoma metastasis, diffuse large B-cell lymphoma or anaplastic large B-cell lymphoma (ALCL).

The presence of atypical cells should cause the pathologist to test for both tryptase and CD117 to identify MCs, which is the major step of the diagnostic process. All of the reported cases were at least CD117 and/or tryptase positive.Thus, immunohistochemistry is strongly recommended to obtain the correct diagnosis of sarcoma mastocytosis. The pathologist plays a key role and should perform the immunohistochemistry when this diagnosis is suspected.

**Figure 2:**
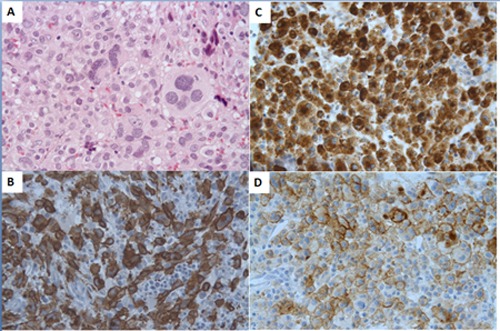
Histological features of mast cells in mast cell sarcoma Staining of a bone marrow biopsy shows the following: **A.** Medium to large pleomorphic mast cells (arrow) with large irregular nuclei, sometimes multinucleated (H&E×400); **B.** Immunostaining with anti-CD117 (KIT) antibody revealing that all atypical large mast cells are strongly positive (×400); **C.** Immunostaining with anti-tryptase antibody showing the same diffuse staining of all atypical mast cells (×400); **D.** Staining with anti-CD30 antibody revealed irregular but strong staining of foci of the atypical mast cells (×400).

### Cytological and immunophenotypic findings

**Figure 3:**
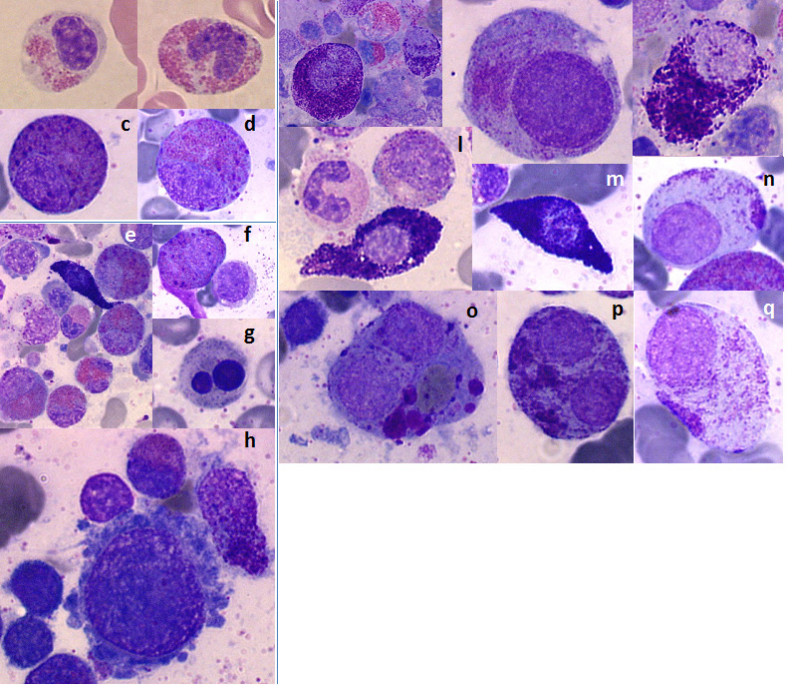
Cytological features from a single case of mast cell sarcoma Left panel: **Blood:** (a, b) Abnormal circulating eosinophils with hypogranular cytoplasm. **Bone marrow:** (c, d) Abnormal bone marrow eosinophils with primary basophil granules. (e) Bone marrow smears at low magnification showing marrow infiltration by eosinophils and mast cells. (f-h) Associated myeloid dysplasia: (f) hypogranular myelocytes and erythroid; (g) binucleated acidophilic erythroblast with abnormal basophils punctuations and megakaryocytic lineages; (h) binucleated acidophilic erythroblast with abnormal basophils punctuations and micromegakaryocyte lineages. - Right panel: **Bone marrow:** (i-j) Hypogranulated mast cells with a visible nucleus. (l-m) Spindle-shaped mast cells. (n-q) Mono- and multinucleated mast cells with coalescent granules resulting in both hypogranular cytoplasm and large compact basophilic granules.

Cytological description of abnormal MCs *in situ* in the initial sarcoma lesion is uncommon given the localized nature of the lesion and the sites of pathological MC proliferation. However, because of the intrinsic propensity of MCS to spread from the initial limited localization to other tissues and in particular to bone marrow, cytological assessment of these cells is feasible in such tissues. A representative case of MCS with extended infiltration to the bone marrow is illustrated in Figure 3.

The cytological features of MCs in MCS are not specific and share multiple aspects and abnormalities with neoplastic MCs found in other subtypes of mastocytosis. Thus, the classical spindle-shaped MCs commonly associated with hypogranular cytoplasm may be observed. In addition, other abnormalities typical of aggressive forms of MC diseases may also be found. Among these, agranular MCs with eccentric and clearly visible nuclei may be observed as well as multinucleated MCs, which have polarized and coalescent granules resulting in a polarized compact basophilic area in the cytoplasm of MCs. Other signs reminiscent of different cell lineages may also be found. Signs of dysplasia involving one of several hematopoietic lineages may be noticed, and occasionally, an excess of (either normal or dysplastic) eosinophils with or without hypereosinophilia in the blood count can be detected.

Finally, in some cases, MCS tends to progress to MCL with neoplastic MCs representing over 20% of the medullar nucleated cells either with (>10% MCs in WBC) or without (<10% MCs in WBC) a leukemic phase.

Likewise, the immunophenotypic characteristics of MCs in MCS are shared with other subtypes of mastocytosis. Neoplastic MCs found in either localized tissue or bone marrow usually present with abnormal expression of CD2 (5/12, 42%) and/or CD25 (9/11, 81%) and are eventually associated with other phenotypic anomalies, especially features of immature MCs, such as weak expression of CD117, in the majority of cases (19/22, 86%).

**Table 2:** Literature review of MCS cases

Authors	Year of publication	No. of cases	Sex	Age at mastocytosis onset (years)	Age at MCS diagnosis (years)	UP pre-existing	Sites of the tumor	KIT mutations	Immunohistochemistry	Leukemic transformation	Survival time (months) Alive£ / deceased
Horny et al [4]	1986	1	F	71	74	No	Subglottic, larynx	NM	NA	Yes	12, Deceased
Sotlar et al [19]	1997	1	M	63	63	No	Retroperitoneal, left testis	NM	CD117+ CD30-	No	NM, Deceased
Kojima et al [20]	1999	1	F	32	32	No	Colon	NM	CD117- Tryp+ CD30-	Yes	24, Deceased
Fine et al [8]	2001	1	M	42	42	No	Choroidal lesion left eye	NM	CD117+ Tryp+	No	>12, NM
Chott et al. [21]	2003	1	F	8	8	No	Temporo-parietal	ND	CD117+ Tryp+ CD2- Ki67 40%	Yes	24, Deceased
Inaoui et al. [9]	2003	1	F	72	72	No	Bones	D816V	CD117+	Yes	>12, Deceased
Brcic et al. [22]	2007	1	M	4	4	No	Left tibia	NM	CD117+ Tryp+ CD2+ Ki67 5%	Yes	12, Deceased
Krauth et al. [15]	2007	1	F	34	34	No	Right femur	ND	CD117+ CD2+ CD25+ Ki67 5%	Yes	36, NM
Bugalia et al. [14]	2011	1	M	71	71	No	Abdominal	Asp 822 Lys (exon 17)	CD117+ Tryp+ CD30-	No	9, Alive
Ma et al.14	2011	1	F	39	39	No	Uterus	ND	CD117- Tryp+ CD2+ CD25+ CD30- Ki67 3%	No	40, Alive
Auquit-Auckbur et al. [10]	2012	1	F	35	35	No	Nodule left ankle	WT	CD117+ Tryp+ CD2+ Ki67 20%	No	36, Deceased
Falleti et al. [23]	2012	1	F	63	63	No	Scalp nodule	D816V	CD117+ Tryp+	No	NM
Georgin-Lavialle et al. [3]	2012	1	M	10	25	Yes	Inguinal	WT	CD117+ Tryp+ CD2- CD25+ CD30+ Ki67 High	No	1, Deceased
Georgin-Lavialle et al. [3]	2012	1	M	42	42	No	Mediastinal infiltrative	Val 560 Gly (exon 11)	CD117+ Tryp+ CD2- CD25-, CD30+ CD52+	No	12, Deceased
Ryan et al. [11]	2012	1	F	12	12	No	Left ear	WT	CD117-Tryp+ CD2- CD25+ CD30- Ki67 5-7%	No	45, Alive
Ryan et al. [11]	2012	1	M	1	19	Yes	Inner lip	Del D419 (exon 8)	CD117+ Tryp+ CD2+ CD25+ CD30+ Ki67 50%	No	19, Alive
Ryan et al. [11]	2012	1	F	77	77	No	Right pelvic	NM	CD117+ Tryp+	No	4, Deceased
Bautista-Quach et al. [24]	2012	1	M	1	1	No	Right external auditory	WT	CD117+ Tryp+ CD2- CD25+ Ki67 1%	No	12, Alive
Kim et al. [13]	2013	1	F	15	15	No	Left temporal bones	Leu 799 Phe (exon 17)	CD117+	No	18, Alive
Schwaab et al. [12]	2013	1	F	69	69	No	Colon	D816V	CD117+ Tryp+ CD25+	No	7, Deceased
CEREMAST # 1	2013	1	M	66	66	No	Bones, digestive	WT	CD117+ Tryp+ CD2- CD25+ CD30+	Yes	4, Alive
CEREMAST # 2	2014	1	M	39	59	No	Bones	WT	CD117+ Tryp+ CD2- CD25- CD30- Ki67 low	No	12, Alive
CEREMAST # 3	2014	1	F	39	39	No	Digestive, Bones	WT	CD117+ Tryp+ CD25+ CD30-Ki67-	No	5, Deceased

Abbreviations: MCS: mast cell sarcoma, F: female; M: male; NM: not mentioned, ND: not detected, WT: wild type

### KIT mutations (Table 1)

Seven exons of the *KIT* gene were sequenced. Exon 17 contained the most frequent mutation, D816V. The other mutations were known to be on exons 8, 9, 10, 11, 12 and 13; therefore, we analyzed exons 8 to 13 to detect the other possible mutations associated with SM [6]. The *KIT* D816V mutation was detected in 3 of 14 (21%) patients tested. Of the remaining 11 patients without *KIT* D816V mutations, 7 patients (50%) had wild-type *KIT* (*KIT* WT), whereas the other 4 patients (29%) had mutations detected in exon 17 (Asp 822 Lys, *n* = 1; Leu 799 Phe, *n* = 1), exon 11 (Val 560 Gly, *n* = 1) and exon 8 (Del D419, *n* = 1). Most of these non-*KIT* D816V mutations have only recently been described, which may explain why older reviews describe *KIT* D816V mutations as the most common. In the *KIT* WT subgroup, 2 patients (33%) had a history of pediatric mastocytosis, whereas none of the 3 patients in the group with positive *KIT* D816V mutation had a pediatric mastocytosis history.

## FDG-PET SCAN IN MCS

**Figure 4:**
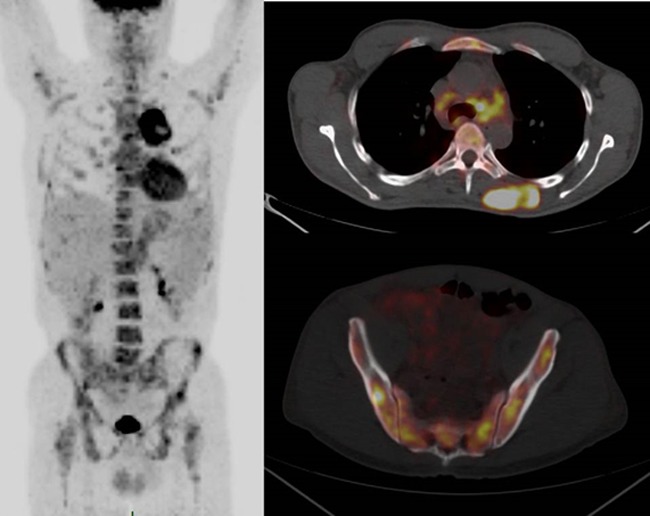
FDG-PET/CT in mast cell sarcoma The sites with confirmed disease are the bone marrow and axial and proximal appendicular skeleton (Figure 4). MIP (maximum intensity projection) image and axial fusion images. Soft tissue mass (dotted arrow) and mediastinal adenopathies (arrowhead) are shown here with multifocal bone marrow uptake.

A recent study from CEREMAST reported on the patterns of FDG-PET/CT in systemic mastocytosis [7]. FDG uptake does not appear to be a sensitive marker of MC activation and proliferation because no significant FDG uptake was observed in most common forms of mastocytosis, notably aggressive SM. However, in this study, all 3 patients with a MCS exhibited a pattern mimicking solid tumor metastasis. The standardized uptake value (SUV) was observed to be intense (range: 3.8-12.2). The sites of disease activity were the bone marrow and axial and proximal appendicular skeleton (Figure 4). These observations suggest a role for FDG-PET as a useful tool for diagnostic and therapeutic assessment in MCS.

## PEDIATRIC MAST CELL SARCOMA (TABLE 1)

Five cases of pediatric MCS have been reported to date. They comprise 3 girls and 2 boys. The mean age at diagnosis of MCS was 8 years. Interestingly, all 5 patients displayed bone involvement, especially in the facial or skull region (4/5 patients; 80%).

Children developed MCS without any previous diagnosis of cutaneous mastocytosis. Interestingly, children presented with bone involvement in 100% of cases whereas the same involvement was observed in only 78% of adults. Furthermore, cranial localization (e.g., temporal bone, ear, lip) occurred in 4 of the pediatric cases. Two of the five patients had wild-type *KIT*, and 3 patients were still alive at more than one year.

Taken together, the results of these 5 sarcoma cases highlight the risk of MCS in children and emphasize the need for *KIT* sequencing despite the rarity of this disease. Based on these cases, we suggest that the structure of the *KIT* gene should be carefully analyzed in children because it may have therapeutic consequences.

**Table 3:** Primary immunohistochemistry characteristics of mast cell sarcoma cells

	CD117 + *N* = 22	CD25 *N* = 11	CD2 *N* = 11	Tryptase *N* = 18	CD68 *N* = 13	CD30 *N* = 12	CD52 *N* = 1	Ki67 *N* = 11
Positive cases; *N* (%)	19 (86)	9 (81)	4 (36)	18 (100)	13 (100)	5 (42)	1 (100)	4 (36)
Negative cases; *N* (%)	3 ()	2 (19)	7 (64)	0	0	7 (58)	0	7 (64)

## DISEASE OUTCOME

MCS evolved into mast cell leukemia in 7 patients (5 adults and 2 children, 30%).

Twelve patients (60%) died, including 10 adults and two children, with a median survival time of 17 months (range 1-45). The median survival time for children and adults was 22.2 (range 12-45) and 15.5 (range 1-40) months, respectively.

The causes of death were MCL (*n* = 7), polymetastatic MCS (*n* = 1), eosinophilic leukemia (*n* = 1), pancytopenia (*n* = 1), bone marrow failure due to myelofibrosis (*n* = 1) and tumor lysis post chemotherapy (*n* = 1). Of the patients tested for *KIT* mutations, death occurred in 2 of 3 patients in the subgroup with the *KIT* D816V mutation and in 5 of 11 patients without any *KIT* mutations.

## MANAGEMENT OF MAST CELL BURDEN

Treatment aims to limit MC burden and increase survival. However, only a few options exist, and the activity of available drugs has been disappointing.

### Surgery

In localized disease, remissions of limited duration have been reported after excision surgery.^8^ However, the best implementation of surgery might be in combination with other treatments such as debulking therapy or in emergency settings such as medullar compression.

### Radiotherapy

Local radiotherapy has been proposed for localized disease. The radiotherapy dosage was variable (40 to 50 Grays), but the efficacy was temporary [4, 8-13]. Interestingly, no acute MCAS have been described following the use of irradiation, but symptomatic therapy could be applied before radiation as a preventive anti-MCAS therapy.

### Corticosteroids

High doses of corticosteroids may induce a reduction in MC burden and improve clinical symptoms, including MCAS. However, this effect is usually transient [CEREMAST experience].

### Interferon-α

In contrast to either ISM or ASM, reports on the use of interferon-α in MCS are scarce. Of the 3 patients with MCS who were treated with interferon-α either with or without prednisone and radiotherapy, only 1 achieved remission for a short duration [9, 14, 15].

### 2-Chlorodeoxyadenosine (2-CdA)

2-CdA as a single agent does not appear to be efficacious against MCS. Of the 3 patients with MCS who received this treatment, none of them achieved remission [3, 12, 15]. Our preliminary experience confirms these reports with solo administration of Cladribine^®^ at a dose of 0.14 mg/kg/day for either 5 or 7 consecutive days [O. Hermine, personal communication]. Combination therapies of 2-CdA with high-dose cytarabine, mTOR inhibitors or TKI thus require further evaluation in patients with MCS.

### Tyrosine kinase inhibitors (TKI) and other targeted therapies

The *KIT* D816V mutant found in adult human mastocytosis causes constitutive activation of the *KIT* kinase. Different classes of *KIT*-activating mutations respond differentially to KIT inhibitors depending on the site and the type of mutation. Particularly, the *KIT* D816V mutation confers resistance to the majority of TKI drugs.

#### Imatinib

In October 2006, the U.S. Food & Drug Administration gave approval for the use of imatinib in adult patients either with ASM lacking the D816V *KIT* mutation or with an unknown *KIT* mutation status at a dose of 400 mg daily.

Six MCS patients treated with imatinib have been reported so far. Five of them had either WT *KIT* or no D816V *KIT* mutations. Responses were partial to complete, with the response duration lasting between 3 and 19 months. Two patients did not respond to imatinib (1 had the *KIT* D816V mutation, and one had *KIT* WT), [10, 11, 14, 16, 17].

#### Dasatinib

Dasatinib has demonstrated significant inhibitory activity at sub-micromolar concentrations *in vitro* against both *KIT* WT and the *KIT* D816V mutation, as well as against juxtamembrane domain mutants of *KIT*. However, its activity has been only modest in clinical settings. Regarding MCS, two patients with *KIT* WT have been treated with dasatinib. Their response was positive; however, the response duration was short [3]. This might be a rationale for developing combination therapies that include dasatinib for patients with the juxtamembrane domain KIT mutation.

#### Midostaurin (PKC412)

PKC412 has shown strong *in vitro* and *in vivo* inhibitory activity on neoplastic human MCs carrying the *KIT* D816V mutation in preclinical and clinical settings.

One MCS patient who received PKC412 in combination with debulking surgery has been described [17]. He obtained complete remission with a response duration of more than 12 months. In our experience, 1 patient with MCS was treated with PKC412 at the dosage of 100 mg b.i.d.; unfortunately, no response was achieved. Thus, combination therapies using PKC412 with either chemotherapy or other targeted therapies might deserve consideration for future evaluations.

### Chemotherapy

Various cytostatic drugs have been shown to induce apoptosis and to inhibit proliferation of the human leukemic MC line HMC-1. However, whether patients with MCS may benefit from combination polychemotherapy remains unknown.

However, the single agent chemotherapies bleomycin, vinblastine and clofarabine [4, 11, 13]; anthracycline-based, cytarabine-based, or ifosfamide plus cyclophosphamide and etoposide combination chemotherapies; or acute myeloid leukemia-type chemotherapies either with or without fludarabine have been reported to exert limited activity in MCS cases [3, 11, 13, 15].

### Bone marrow transplantation

Allogeneic stem cell transplantation (Allo-SCT) could be a potential curative treatment in patients with aggressive systemic mastocytosis [18]. However, due to the absence of sufficient data from MCS patients, allo-SCT remains untested, and further evaluation of this strategy in such patients is necessary.

## MATERIALS AND METHODS

### Bibliographical search strategy

Two electronic databases, Medline via PubMed and the Embase Library, were searched using a MeSH and text terms for “sarcoma,” “cutaneous mastocytosis,” systemic mastocytosis,” “mast cell sarcoma,” and “mast cell leukemia.” The search was restricted to human cohorts published in English and French between 1986 and May 2014. Furthermore, references of included studies and relevant review articles were scanned to identify additional studies. Published full text articles were systematically selected. We excluded manuscripts that were only presented in abstract form or for which full text was unavailable. In total, 20 articles with full text were available. Only cases that met the WHO criteria for the diagnosis of MCS and/or systemic mastocytosis were included (Table 1).

We included additionally 3 unpublished French cases from the National Reference Center for Mastocytosis [CEREMAST].

### Data extraction and analysis

The following data from each article were extracted by 2 independent authors (JM, SGL): patient demographics (e.g., age, sex, geographic origin when available, personal or familial history of mastocytosis, age at disease onset), disease characteristics (e.g., date of diagnosis, time from the start of symptoms to diagnosis, organ involvement, cutaneous lesions, localization of the sarcoma, mast cell activation symptoms (MCAS)), biological characteristics (e.g., blood cell counts, serum tryptase level, MC phenotype, *KIT* mutations), histopathology, cytology and disease and patient outcome. The data analysis and verification were performed by three authors (JM, SGL, GD).

## CONCLUSION

MCS is a rare form of highly aggressive systemic mastocytosis with poor prognosis and few therapeutic options. It primarily manifests *de novo* but can also appear following a preexisting different subtype of mastocytosis. This type of sarcoma frequently involves bones and the digestive tract. Serum tryptase levels are usually high, and MCs are commonly CD2 and/or CD25 positive. Wild-type or *KIT* D816V mutations are frequent, and complete gene sequencing is needed. Therapy usually fails, which results in a short median survival time. Thus, new combination therapies that include *KIT* inhibitors, chemotherapy, 2-CdA, anti-CD30 and bone marrow transplantation should be evaluated to treat this devastating disease.
